# Estimation of (co)variances for genomic regions of flexible sizes: application to complex infectious udder diseases in dairy cattle

**DOI:** 10.1186/1297-9686-44-18

**Published:** 2012-07-06

**Authors:** Lars P Sørensen, Luc Janss, Per Madsen, Thomas Mark, Mogens S Lund

**Affiliations:** 1 University of Aarhus, Faculty of Science and Technology, Department of Molecular Biology and Genetics, DK-8830, Tjele, Denmark; 2 University of Copenhagen, Faculty of Life Sciences, Quantitative and Systems Genetics Group, DK-1870, Frederiksberg C, Denmark

## Abstract

**Background:**

Multi-trait genomic models in a Bayesian context can be used to estimate genomic (co)variances, either for a complete genome or for genomic regions (e.g. per chromosome) for the purpose of multi-trait genomic selection or to gain further insight into the genomic architecture of related traits such as mammary disease traits in dairy cattle.

**Methods:**

Data on progeny means of six traits related to mastitis resistance in dairy cattle (general mastitis resistance and five pathogen-specific mastitis resistance traits) were analyzed using a bivariate Bayesian SNP-based genomic model with a common prior distribution for the marker allele substitution effects and estimation of the hyperparameters in this prior distribution from the progeny means data. From the Markov chain Monte Carlo samples of the allele substitution effects, genomic (co)variances were calculated on a whole-genome level, per chromosome, and in regions of 100 SNP on a chromosome.

**Results:**

Genomic proportions of the total variance differed between traits. Genomic correlations were lower than pedigree-based genetic correlations and they were highest between general mastitis and pathogen-specific traits because of the part-whole relationship between these traits. The chromosome-wise genomic proportions of the total variance differed between traits, with some chromosomes explaining higher or lower values than expected in relation to chromosome size. Few chromosomes showed pleiotropic effects and only chromosome 19 had a clear effect on all traits, indicating the presence of QTL with a general effect on mastitis resistance. The region-wise patterns of genomic variances differed between traits. Peaks indicating QTL were identified but were not very distinctive because a common prior for the marker effects was used. There was a clear difference in the region-wise patterns of genomic correlation among combinations of traits, with distinctive peaks indicating the presence of pleiotropic QTL.

**Conclusions:**

The results show that it is possible to estimate, genome-wide and region-wise genomic (co)variances of mastitis resistance traits in dairy cattle using multivariate genomic models.

## Background

Livestock provide a great source of data to investigate genome-wide effects on various phenotypic characteristics such as infectious diseases. There are several reasons for this, including: (1) vast amounts of phenotypic measures (milk yield in dairy cattle, litter size in pigs, daily gain in broilers etc.) are systematically recorded in modern livestock production and in Danish dairy cattle, for example, phenotypic information on a variety of traits, including clinical disease, is stored together with pedigrees in one central database; (2) important environmental factors, such as herd membership, affecting various phenotypes are recorded and animals within such groups receive rather homogeneous treatments; (3) low effective population sizes are frequent in livestock (e.g. compared with humans), which makes it easier to predict genetic merit and (4) recently, routine genotyping using dense SNP marker panels (e.g. >50 K) for thousands of animals has been initiated in several livestock species.

In the Nordic countries (Denmark, Finland, Norway, and Sweden), treatment of udder infections (mastitis) in dairy cattle is systematically recorded by veterinarians or farmers. However, estimates of heritability of mastitis incidence are low (i.e. 0.1 on the underlying continuous scale or 0.03 on the observable scale; [1] and [2], respectively). The disease can be caused by a large number of microbial pathogens [3], which differ in pathogenesis and reservoir. Several studies have shown that the mammary immune response differs between pathogens [4,5] suggesting that it is regulated by different genes and that mastitis caused by different pathogens should be considered as different traits. This is supported by our previous study [1] in which pedigree-based analyses were conducted to estimate genetic correlations between mastitis caused by different pathogens. The genetic correlations between mastitis caused by five common mastitis pathogens, *Staphylococcus aureusEscherichia coli*, coagulase-negative staphylococci (CNS), *Streptococcus dysgalactiae*, and *Streptococcus uberis*, ranged from 0.45 to 0.77, which implies that the mammary immune system, or the physical defense system, or both, act in a pathogen-specific manner. However, the existence of positive genetic correlations also implies the presence of pleiotropic effects or linked quantitative trait loci (QTL). Several studies have reported different heritability estimates for pathogen-specific mastitis traits [6-8], indicating that they may differ between traits, although some of these differences may also be due to differences in data structure and in the method used to estimate genetic parameters.

Genomic data are now used to infer either (1) whole-genome effects for the purpose of, e.g., estimation of breeding values to select superior breeding animals or for prediction of future phenotypes such as disease risks, or (2) effects of single genes or markers, to guide the development of human or veterinary drugs through improved knowledge on the biological basis of traits. Approach (1) typically involves ‘whole genome’ models that model all SNP simultaneously, whereas approach (2) involves Genome-Wide Association Studies (GWAS), in which, typically, each SNP is tested individually using univariate association tests. Here, we suggest a compromise between these two approaches by employing whole-genome models in which variances and covariances are partitioned by chromosome segments. We hypothesize that this approach will capture a large portion of the genetic variance, while also providing further biological understanding of the traits in question. Investigating the effects of chromosome segments of variable size (e.g. regions of neighboring SNP, haplotypes, gene-networks, chromosomes) and correlations among segment effects on different traits may provide interesting insights into the genetic and biological architecture of disease traits such as mastitis incidence.

Statistical methods for genomic analyses typically employ fixed prior parameters, which make them less suited to estimate genomic (co)variances. Models that use a genomic relationship matrix, e.g. [9], could be used to estimate (co)variances using REML (Restricted Maximum Likelihood) but studying (co)variances per chromosome or for several chromosome segments would be computationally prohibitive. For instance, a bivariate analysis in dairy cattle with 30 chromosomes would involve 30 genomic relationship matrices and the simultaneous estimation of 90 variance-covariance components. Using multivariate genomic selection methodology [10] for mastitis traits, it is possible to build a (co)variance matrix of allele substitution effects. In this study, we used a Bayesian SNP-based genomic model, which was extended to estimate hyperparameters of the prior distribution of allele substitution effects from the data. Thereby, the method makes it possible to estimate genomic (co)variances while remaining computationally feasible. Results can be used to reveal genomic regions associated with only one pathogen (pathogen-specific effects), associated with two or more pathogens (group-specific effects), or associated with all the pathogens (general effects). The estimated (co)variances between the allele substitution effects can also be used to compute various genetic parameters such as heritabilities and correlations; these can be computed region-wise (e.g. per chromosome) or genome-wide.

The objectives of this work were to (1) present a multivariate model for genome-wide and region-wise association studies, (2) perform simultaneous estimation of genomic effects (allele substitution effects) for mastitis resistance using more than one trait, and (3) estimate covariances between traits across the chromosomes and across regions of various sizes.

## Methods

### Phenotypic data

The data comprised records of mastitis treatments and pathogen information (results of bacteriological culturing of milk samples) from Danish Holstein cows that calved for the first time between January 1998 and January 2009 (collection period). The data were extracted from the Danish National Cattle Database. Mastitis is a difficult trait to analyze due to its low heritability and a potential bias in the treatment of cows; thus, data were edited as described in [11]. Briefly, data from cows that had calved after March 2008 (300 days before the end of the collection period) were removed from the dataset to reduce the bias due to censoring. In addition, the following criteria were required for a herd to be included in the dataset: age at first calving between 19 and 36 months for a cow to be included in the data set, participating herds with at least 30 first calvings in a given year of the collection period, and active participation in disease recording [12]. Information on mastitis treatments was merged with pathogen data if the recorded date of a pathogen was three days before to four days after a case of mastitis was recorded on the same cow. Only the data from daughters of genotyped bulls were included in the present study and each bull was required to have at least five daughters calving during the collection period, resulting in a dataset of 200 149 daughters of 1 844 genotyped sires.

### Trait definitions

General mastitis was defined as a binary trait for the period from15 days before to 300 days after first calving, i.e. a pheno type of “1” was assigned if a cow was treated for mastitis during this period and “0” otherwise. Only the first observed mastitis treatment for each cow was included. The five most common pathogens in Danish dairy herds, i.e. *Staph. aureus*, CNS, *E. coli*, *Strep. dysgalactiae*, and *Strep. uberis*, were chosen to represent the pathogen-specific mastitis traits (also binary). The pathogen-specific traits were defined only for treatments with pathogen information. In contrast, the trait “general mastitis” contained all recorded (according to trait definition) treatments of mastitis, i.e. both treatments with and without pathogen information.

### Estimation of progeny means (PM) adjusted for non-genetic effects

For computational reasons, it was necessary to summarize information per sire (meta-analysis) due to the small number of sires and the large number of offspring per sire, and availability of SNP information on the sires only. Thus, PM of the mastitis traits were estimated as daughter yield deviations, as described by [13]. However, in the present study, a sire model was used to estimate both PM and EBV; thus PM were defined as a trait corrected for all known environmental effects and averaged over records so that they consisted of additive genetic and residual effects. For the mastitis traits, a threshold-liability model [14] was applied to estimate PM. The threshold model assumes the presence of an underlying continuous random variable called liability, *λ*. The relationship between the observed binary variable, *y,* and the unobservable *λ* is

(1) yi={0ifλi≤τ1ifλi>τ

where *τ* is a fixed threshold and *y*_*i*_ = 1 and 0 correspond to the presence or absence of mastitis for observation *i*, respectively. It was assumed that *λ* is normally distributed with a mean vector *μ* and covariance matrix $R=I\sigma_{e}^{2}$. Because *τ* and $\sigma_{e}^{2}$ are undetermined, they were arbitrarily set equal to *τ* = 0 and $\sigma_{e}^{2}$ = 1 such that

(2) $$\lambda |\mu ∼\sim N\;\left(\mu ,I\right)$$

The probability (*π*_*i*_) that observation *i* is scored as “1” given the model parameter vector **θ**, is

(3) $$\begin{array}{c} \;\pi_{i}=Pr\;\left(y_{i\;}=1|\theta \right)\\ =Pr\;\left(\lambda_{i}>0|\theta \right)\\ =1-Pr\;\left(\lambda_{i}\leq 0|\theta \right)\\ =\Phi \;\left(\mu_{i}\right) \end{array}$$

where Φ(.) is the standard normal cumulative distribution.

The following sire model was used to describe liability to mastitis:

(4) $$\lambda_{\mathrm{ijklm}}=YM_{i}+AGE_{j}+b_{1}\;t_{\mathrm{ijklm}}+hys_{k}+sire_{l}+e_{\mathrm{ijklm}}$$

where

*λ*_*ijklm*_ = liability to mastitis of daughter *m* of sire *l* calving in year-month class *i* at calving age class *j* and in herd-year-season class *k*;

*YM*_*i*_ = “fixed” effect of year-month of calving (123 classes);

*AGE*_*j*_ = “fixed” effect of calving age (17 classes);

*hys*_*k*_ = random effect of herd-year-season (season = year divided into quarters; 22,918 levels);

*sire*_*l*_ = transmitting ability of sire *l* (8 547 levels);

*b*_1_ = “fixed” regression coefficient of *λ* on the length of the period at risk;

*t*_*ijklm*_ = period at risk for daughter *m* of sire *l*, defined as the number of days from 15 days before calving to the date of culling or to the end of the risk period; it was assumed that all cows with mastitis had a completed risk period;

*e*_*ijklm*_ = residual ~ *N*(0,1) and independent.

In matrix notation, the model for the mastitis traits can be expressed as:

(5) $$\lambda =X_{b}b+X_{h}h+Zs+e$$

where **λ** is a *n* × 1 vector of the underlying liabilities of mastitis, *n* is the number of records for each trait, **b** is a vector of “fixed” effects as described previously, **h** is a vector of random herd-year-season effects, **s** is a vector of random sire effects, and **e** is a vector of random residual effects. **X**_b_, $X_{h_{i}}$ and **Z** are corresponding incidence matrices.

A full Bayesian approach using Markov chain Monte Carlo (MCMC) methods [15] via Gibbs sampling implemented in the DMU package [16] was used to fit the models and sample posterior PM. The PM were on the liability scale and were estimated from the model above as $PM_{i}=\sum_{k}TD_{k}/n$, where TD_*k*_ is the trait of daughter *k* on the liability scale and adjusted for all effects other than additive genetic effects and residuals and *n* is the number of daughters of bull *i*. Independent improper uniform priors were assigned to each element of **b**. Herd and sire effects were assigned uninformative normal prior distributions $h∼N\left(0,I\sigma_{h}^{2}\right)$and $s∼N\left(0,A\sigma_{s}^{2}\right)$, respectively, where **I** is an identity matrix, **A** is the additive relationship matrix, and $\sigma_{h}^{2}$ and $\sigma_{s}^{2}$ are the herd and sire variances, respectively. Independent scaled inverse chi-square distributions were used for the unknown variance components ($\sigma_{h}^{2}$ and $\sigma_{s}^{2}$), with settings so that these prior distributions were flat. Inferences were based on 600 000 samples; the first 100 000 samples were disregarded as burn-in, and every 10^th^ sample was saved for post-Gibbs analyses.

Convergence of the Gibbs chains for each model parameter was ensured using a standardized time series method of batch means [17,18].

### Estimation of heritabilities of progeny means

Subsequently, genetic variances of the estimated PM were estimated using a standard linear animal model with pedigree information and REML. The PM were weighted based on the standard errors of prediction (SEP) of the posterior PM samples. From the estimated variances, heritabilities for each trait PM were computed for later comparisons with estimated genomic variances.

### Weights for the association model

Standard errors of prediction of the posterior PM samples were calculated to construct weights for each trait included in the genomic model to adjust for heterogeneous variances of the sire records. The weights were computed as 1/SEP^2^ and scaled to achieve an average weight of 1. The scale factor used in the present study was the average weight per trait of the 1 844 genotyped bulls. By scaling the weights to an average of 1, the computed residual variances will be directly comparable with the genomic (co)variances.

### Marker data

The bulls selected for this study were genotyped using the Illumina Bovine SNP50 BeadChip (Illumina, San Diego, CA). The raw marker data were edited using the following criteria: (1) a locus was removed from the analyses if the minor allele frequency was less than 5%, if the proportion of animals genotyped for this locus was less than 95%, if the average GenCall score at the locus was less than 60%, and if the proportion of missing marker genotypes was larger than 10%; (2) an individual was deleted if the call rate (i.e. the overall call rate of a sample is equal to the number of SNP receiving an AA, AB, or BB genotype call divided by the total number of SNP on the chip) had a score below 0.85. After editing, 1 844 bulls had daughters with mastitis and pathogen data, and 37 862 SNP were available and used in the analyses.

### Genomic model

Genomic parameters were estimated using a Bayesian model in which SNP effects, within a trait, were assumed to originate from the same normal distribution. This represents the gBLUP method [9] implemented with Bayesian methodology [19] and a random walk Metropolis-Hastings algorithm to obtain MCMC samples for variance components [20]. The difference between the method described in [9] and the present method is that the variances are also treated as unknown model parameters in the Bayesian model, so that variances and SNP effects are jointly estimated in a single model. This allows for estimation of individual SNP effects, which allows the model to be more easily scaled up to a multi-trait analysis. Weighted residuals were used in the model and latent variables were used to model the covariances between traits within each SNP and between residuals. The bivariate model specification was:

(6) $$\left\{ \begin{array}{c} PM_{1}=1\mu_{1}+\sum_{i=1}^{M}X_{i}b_{1i}+v_{1}\;W_{1}^{-1/2}1+e_{1}\\ PM_{2}=1\mu_{2}+\sum_{i=1}^{M}X_{i}b_{2i}+v_{2}\;W_{2}^{-1/2}1+e_{2} \end{array}\right.$$

where PM_1_ and PM_2_ are vectors with PM for the two traits on a common list of individuals, *μ*_1_ and *μ*_2_ are the PM means of each trait, **x**_*i*_ are vectors of coded genotypes of the individual for *i* = 1, …, *M* SNPs, *b*_k*i*_ is the random regression coefficient modeling the effect for SNP *i* on trait *k***W** is a diagonal matrix with 1/SEP^2^ as diagonal elements, **l** is a vector of latent effects that models the correlated part of the residuals (note the use of the same vector **l** for both traits), *ν*_1_ and *ν*_2_ are scale factors for the effect of the latent vector **l** on each trait, which can be interpreted as the elements of the first eigenvector of the residual variance-covariance matrix (see below), and *e*_1_ and *e*_2_ are the uncorrelated parts of the model residuals.

The genotype coding in *x*_i_ was done as 2*p*-2, 2*p*-1, and 2*p* for homozygotes for the first allele, heterozygotes, and homozygotes for the second allele. This is similar to [21], except that *p* is the frequency of the first allele. Such coding standardizes the means of the genotype covariates to zero, assuming Hardy-Weinberg equilibrium of genotype frequencies, and the regression of such a genotype coding on the PM represents the allele substitution effect for substituting the first coded with the second coded allele. Covariances between the SNP effects were also modeled using a latent variable, but this was specified as a hierarchy in the Bayesian model. In this multi-trait model, the effects of a SNP on the two traits were correlated; therefore the variance of marker and residual effects were $var\;\left(b_{1},b_{2}\right)∼\left[\begin{array}{cc} \sigma_{b_{1}}^{2} & \sigma_{b_{1}b_{2}}\\ \sigma_{b_{1}b_{2}} & \sigma_{b_{2}}^{2} \end{array}\right]$ and $var\;\left(e_{1},e_{2}\right)∼\left[\begin{array}{cc} \sigma_{e_{1}}^{2} & \sigma_{e_{1}e_{2}}\\ \sigma_{e_{1}e_{2}} & \sigma_{e2}^{2} \end{array}\right]$, respectively. Note that the elements of *var*(*b*_1_*b*_2_) were assumed the same across the genome.

The distributional assumptions of the model parameters were:

(7) $$\begin{array}{l} \begin{array}{c} \begin{array}{c} \begin{array}{c} \mu_{1}\text{,}\;\mu_{2}\text{,}\;v_{1}\text{,}\;v_{2}∼U\;\left(-\infty \text{,}\;\infty \right)\\ 1∼N\;\left(0\text{,}\;I\delta_{1}^{2}\right)\\ e_{1}∼N\;\left(0\text{,}\;W_{1}^{-1}\;\delta_{2}^{2}\right) \end{array}\\ e_{2}∼N\;\left(0\text{,}\;W_{2}^{-1}\;\delta_{2}^{2}\right)\\ b_{1}∼N\;\left(u_{1}s\text{,}\;t_{2}^{2}\right) \end{array}\\ b_{2}∼N\;\left(u_{2}s\text{,}\;t_{2}^{2}\right)\\ s∼N\;\left(0\text{,}\;It_{1}^{2}\right) \end{array}\\ \begin{array}{c} \delta_{1}^{2}\text{,}\;\delta_{2}^{2}\text{,}\;t_{1}^{2}\text{,}\;t_{2}^{2}∼U\;\left(0\text{,}\;\infty \right)\\ u_{1},u_{2}∼U\;\left(-\infty \text{,}\;\infty \right) \end{array} \end{array}$$

with the constraint that |*ν*| = 1, where *ν* = (*ν*_1_, *ν*_2_), |*u*| = 1, where *u* = (*u*_1_,*u*_2_), and where *N*() denotes a normal distribution with mean and variance parameter, *U*() denotes a uniform distribution on the given interval.

The modeled residual variance-covariance structure can be shown to be:

(8) $$\begin{array}{l} \text{var}\left(\begin{array}{l} \nu_{1}W_{1}^{-1/2}l+e_{1}\\ \nu_{2}W_{2}^{-1/2}l+e_{2} \end{array}\right)=\left[\begin{array}{cc} \nu_{1}^{2}W_{1}^{-1}\delta_{1}^{2}+I\delta_{2}^{2} & \nu_{1}\nu_{2}W_{1}^{-1/2}W_{2}^{-1/2}\delta_{1}^{2}\\ \nu_{2}\nu_{1}W_{2}^{-1/2}W_{1}^{-1/2}\delta_{1}^{2} & \nu_{2}^{2}W_{2}^{-1}\delta_{1}^{2}+I\delta_{2}^{2} \end{array}\right]\\ =\left[\begin{array}{cc} \nu_{1}^{2}\delta_{1}^{2}+I\delta_{2}^{2} & \nu_{1}\nu_{2}\delta_{1}^{2}\\ \nu_{2}\nu_{1}\delta_{1}^{2} & \nu_{2}^{2}\delta_{1}^{2}+I\delta_{2}^{2} \end{array}\right]\times \left[\begin{array}{cc} W^{-1} & W_{1}^{-1/2}W_{2}^{-1/2}\\ W_{2}^{-1/2}W_{1}^{-1/2} & W_{2}^{-1} \end{array}\right] \end{array}$$

where the first part corresponds to a special form of the spectral decomposition of the variance-covariance matrix **R**, such that it can be shown that *ν* = (*ν*_1_, *ν*_2_) is the first eigenvector of **R**, $\delta_{2}^{2}$ is the second eigenvalue of **R**, and $\delta_{1}^{2}$ estimates the difference between the first eigenvalue and the second eigenvalue. In the same way, the vectors of SNP effects, **b**, are correlated through the use of common latent vectors, **s**, and the variance-covariance structure for SNP effects can be shown to have a covariance of $u_{1}u_{2}t_{1}^{2}$ and variances $u_{1}^{2}t_{1}^{2}+t_{2}^{2}$ and $u_{2}^{2}t_{1}^{2}+t_{2}^{2}$. Again, (*u*_1_,*u*_2_) can be interpreted as the first eigenvector of the variance-covariance matrix, $t_{2}^{2}$ as the second eigenvalue, and $t_{1}^{2}$ as the difference between the first eigenvalue and the second eigenvalue.

#### Implementation

The MCMC estimation for this model was straightforward for *μ*_1_, *μ*_2_, *b*_1*i*_, *b*_2*i*_, because these parameters have conditional normal distributions that are independent between traits and therefore can be updated in a “single trait manner”. Also *ν*_1_ and *ν*_2_ have conditional normal distributions and were updated as a regression on the vector **l**. They were scaled to unity norm after sampling to apply the constraint on |*ν*|. The same inverse scaling was applied to the latent vector **l**, because *ν* and **l** are multiplicative in the model. Applying the same scaling to both *ν* and **l** is arbitrary but forces the model to uniquely explain all the variance through **l** and makes parameters identifiable. The vector of latent residual effects, **l**, works across traits but its conditional distribution is also normal and derived by unifying the two trait equations into a single equation:

(9) $$\left[\begin{array}{c} {\tilde{y}}_{1}\\ {\tilde{y}}_{2} \end{array}\right]=\left[\begin{array}{c} W_{1}^{-1/2}v_{1}\\ W_{2}^{-1/2}v_{2} \end{array}\right]\;\left[1\right]+\left[\begin{array}{c} e_{1}\\ e_{2} \end{array}\right]\text{,}$$

where ${\tilde{y}}_{k}=y_{k}-\mu_{k}-\sum_{i=1}^{M}x_{i}b_{\mathrm{ki}}$

#### Posterior analyses

Model fit was assessed by visual inspection of model residuals plotted against the reliability of the estimated breeding values (EBV) for the six traits and significance of the slope of regression line from zero was tested using a t-test. Reliability of EBV was calculated as:

(10) $$r^{2}=\left(1-\left(\frac{SEP^{2}}{\sigma_{s}^{2}}\right)\right)$$

Posterior statistics for any function of the model parameters can be easily obtained when such a function is computed on the primary MCMC samples of the model parameters. This was applied to compute direct genomic breeding values (DGV) of individuals and genomic and residual (co)variances per chromosome and parameters derived thereof. For all these estimates, posterior means and posterior standard deviations were obtained. The genomic parameters were based on the constructed DGV of individuals which automatically take into account the covariance generated between SNP due to linkage disequilibrium (LD). Markov chain Monte Carlo samples of individual genomic values for trait 1 ($g_{1}^{*}$) and trait 2 ($g_{2}^{*}$) were constructed from the MCMC samples of allele effects for the two traits ($b_{1}^{*}$,$b_{2}^{*}$) as $g_{1}^{*}=\sum x_{i}b_{1i}^{*}$ and $g_{2}^{*}=\sum x_{i}b_{2i}^{*}$. Using markers only in specified intervals (e.g. per chromosome or specified blocks of SNP within a chromosome), MCMC samples of individual DGV per interval $g_{1c}^{*}$ and $g_{2c}^{*}$ for interval *c* were constructed. From the MCMC samples of individual DGV, MCMC samples of genomic variances and covariances were subsequently constructed by computing $\sigma_{g1}^{2}*=var\;\left(g_{1}^{*}\right)$, $\sigma_{g2}^{2}*=var\;\left(g_{2}^{*}\right)$, and $\sigma_{g12}*=cov\;\left(g_{1}^{*}\text{,}\;g_{2}^{*}\right)$_,_ which was done for the whole-genome DGV and for the interval-wise DGV. Furthermore, MCMC samples of genomic correlations, $r_{g}=\frac{\sigma_{g12}^{*}}{\sigma_{g1}^{*}.\sigma_{g2}^{*}}$, were computed, and finally MCMC samples of the genomic proportions of the total variance (GPV) were computed. From these constructed MCMC samples, posterior statistics such as the posterior means and posterior standard deviations were collected.

Inferences were based on 40 000 samples with a burn-in of 5 000 samples. Every 50^th^ sample was saved and used for post-MCMC analysis. Convergence of the Markov chains was ensured by visual inspection of trace plots and plots of autocorrelations between lags for each model parameter.

## Results

**Figure 1:**
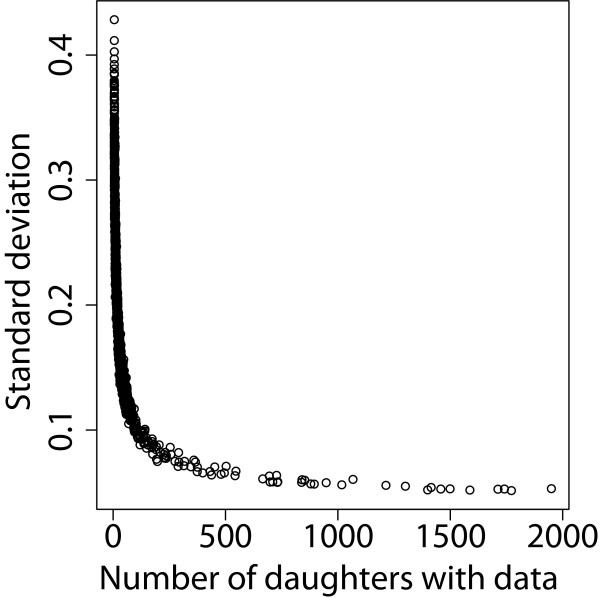
**Relationship between number of daughters and standard deviations of progeny means for general mastitis.** Standard deviations for bulls with more than 2 000 daughters with data are not shown.

The number of daughters with data from individual bulls and the low heritabilities of the traits both affected the reliability of the EBV and the posterior standard deviations of the PM. For example, 80% of the bulls had between 5 and 50 daughters with phenotypic information. This resulted in average reliabilities of EBV of 0.30, 0.36, 0.32, 0.30, and 0.38 for mastitis caused by *Staph. aureus*, CNS, *E. coli*, *Strep. dysgalactiae*, and *Strep. uberis*, respectively. The heritability of general mastitis was higher than that of the pathogen-specific mastitis traits, resulting in a higher average reliability, i.e. 0.57. Accuracy of the PM was assessed by studying their posterior standard deviations (SD). Figure 1 shows that the SD for general mastitis was larger when the number of daughters was low. Similar results were observed for the pathogen-specific mastitis traits (not shown).

### Model fit

**Figure 2:**
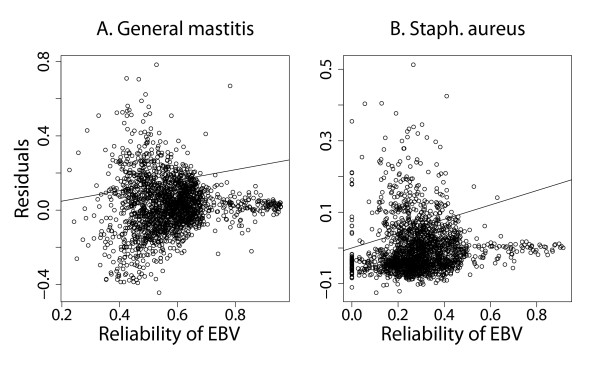
**Examples of model residuals**^**a**^** against reliability of estimated breeding values for general mastitis (A) and mastitis caused by *Staph. aureus* (B).**^a^Observed progeny means minus predicted direct genomic values.

Model fit was assessed by plotting model residuals (observed PM-DGV) against different variables. In Figure 2, examples of plots of residuals against reliability of EBV are shown for general mastitis and mastitis caused by *Staph. aureus*. The slope of the regression line was significantly different from zero (t-test; *p* < .0.05) for all traits except *Staph. aureus* mastitis. For *Staph. aureus* mastitis, the estimation errors of the DGV clearly increased when reliabilities of the EBV reached values below 0.5. This trend was observed for all the pathogen-specific mastitis traits. For general mastitis, which had higher heritability and EBV reliabilities estimation errors of the DGV increased when EBV reliabilities were below 0.7.

### Whole-genome GPV

The average of the posterior means of GPV from the pair-wise analyses of the mastitis traits differed among traits (Table 1). Among the pathogen-specific mastitis traits, the largest value was found for CNS, followed by *Strep. uberis*, *E. coli*, *Staph. aureus*, and *Strep. dysgalactiae*. Analysis of a trait in different pair-wise trait combinations, resulted in similar GPV for the trait. As expected, the GPV of general mastitis was higher than that of pathogen-specific mastitis traits, except when compared to mastitis caused by CNS. Table 1, shows the pedigree-based heritabilities of the trait PM for comparison. Pedigree-based heritabilities were all smaller than the GPV but had the same ranking across traits.

**Table 1:** **Whole-genome genomic proportions of total variance (GPV) and pedigree-based estimates of heritability *h***^**2**^** for progeny means of mastitis susceptibility to five pathogens and general mastitis and standard deviations (SD) of the estimates**

**Trait**	**GPV**^**a**^**(range)**	**Average GPV**^**b**^	**SD (range)**	***h***^**2**^
*Staph. aureus*	0.46-0.48	0.48	0.022-0.025	0.58
CNS	0.62-0.64	0.63	0.020-0.023	0.79
*E. coli*	0.47-0.48	0.47	0.024-0.026	0.56
*Strep. dysgalactiae*	0.40-0.42	0.41	0.024-0.029	0.50
*Strep. uberis*	0.49-0.51	0.51	0.023-0.025	0.59
General mastitis	0.52-0.52	0.52	0.024-0.026	0.64

Pedigree based heritabilities are shown for comparison; ^a^from multiple pair-wise combinations with the trait; ^b^average across analyses

### Whole-genome correlation

Genomic correlations (Table 2) among the investigated traits were moderate to high (0.22 to 0.72). Genomic correlations among the pathogen-specific traits (0.22 to 0.51) were lower than genomic correlations between general mastitis and the pathogen-specific mastitis traits (0.55 to 0.72) because of their part-whole relationship, i.e. pathogen-specific cases of mastitis are part of the general mastitis cases.

**Table 2:** Genomic correlations among the five pathogen-specific mastitis traits and general mastitis

**Trait**^**a**^	**CNS**	**COL**	**DYS**	**UBE**	**MAS**
AUR	0.22 (0.04)	0.28 (0.04)	0.42 (0.05)	0.25 (0.05)	0.55 (0.03)
CNS		0.37 (0.05)	0.38 (0.04)	0.51 (0.04)	0.62 (0.03)
COL			0.32 (0.05)	0.39 (0.04)	0.67 (0.03)
DYS				0.45 (0.05)	0.61 (0.04)
UBE					0.72 (0.03)

Posterior standard deviations in brackets; ^a^AUR: *Staph. aureus*; CNS: coagulase-negative staphylococci; COL: *E. coli*; DYS: *Strep. dysgalactiae*; UBE: *Strep. uberis*; MAS: general mastitis

### Chromosome-wise GPV

**Figure 3:**
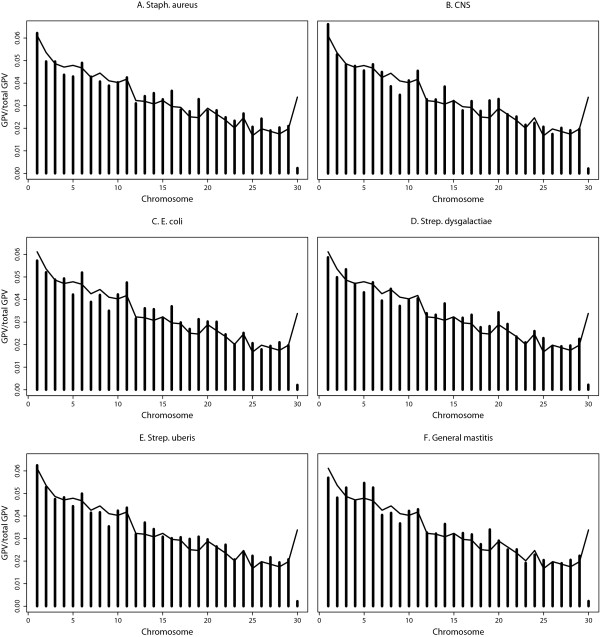
**Chromosome-wise genomic proportions of total variance relative to the total size of the genome.** Bars reaching above the line indicate that the chromosome explains more variance than expected based on its size; GPV as bars and relative genome size as line; pathogen-specific traits, **A-E**, and general mastitis, **F**.

All chromosomes explained significant amounts of genetic variance for each trait, except chromosome X (BTA30), which explained substantially less variance than would be expected based on its size. For example, the variance explained by chromosome X was 93% lower than expected for general mastitis. For the other chromosomes, the trend is that chromosome-wise GPV (Figure 3) increases with chromosome size (*Bos Taurus* 4.0; [22]), i.e. larger chromosomes tend to explained more genomic variance. This pattern was seen for all traits.

Some chromosomes deviated from the general trend and explained more variance than would be expected according to their relative size, i.e. they may contain relatively more QTL or QTL with larger effects on the trait and such chromosomes differed across traits; chromosomes with relatively large GPV were BTA6, 13, 14, 16, 19, and 26 for *Staph. aureus* mastitis; BTA1, 11, 14, 17, 19, and 20 for CNS; BTA6, 11, 13, 14, 16, 19, and 21 for *E. coli*; BTA3, 14, 17, 19, 20 and 25 for *Strep. dysgalactiae*; and BTA6, 13, 14, 18, 19, 25 and 27 for *Strep. uberis*. Additional chromosomes with less pronounced effects, but still above their expected value, were observed for most of the pathogen-specific traits. Chromosomes showing a large variance for general mastitis were BTA3, 5, 6, 14, and 19. Only BTA19 had higher GPV than expected according to size for all traits.

### Chromosome-wise genomic covariances

**Figure 4:**
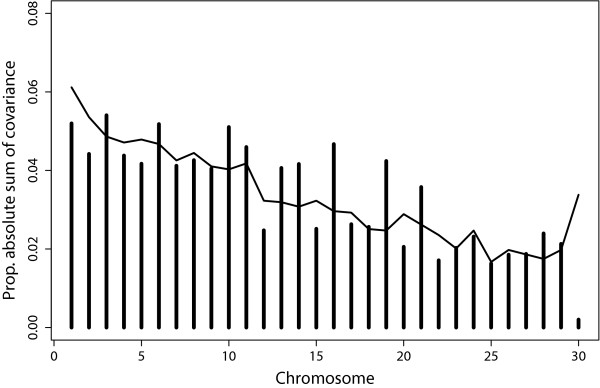
**Chromosome-wise genomic proportions of the sum of covariance**^**a**^** between *Staph. aureus *and *E. coli.*** Bars reaching above the line indicate that a chromosome explains more genomic covariance than expected based on its size; ^a^negative covariances were not observed; covariances as bars and relative genome size as line.

For all chromosomes, covariances between traits were positive (results not shown), resulting in overall positive genomic correlations between the traits, given the default prior assumptions for the latent vector, **l**. For all trait pairs, there was a high proportion of chromosomes with larger covariances than expected, which may indicate the presence of pleiotropic QTL. In addition, in several cases a limited number of chromosomes accounted for a major part of the total covariance, e.g. BTA3, 6, 10, 11, 13, 14, 16, 19, 21 and 29 for the covariance between *Staph. aureus* and *E. coli* (Figure 4).

### Chromosome-wise genomic correlations

**Figure 5:**
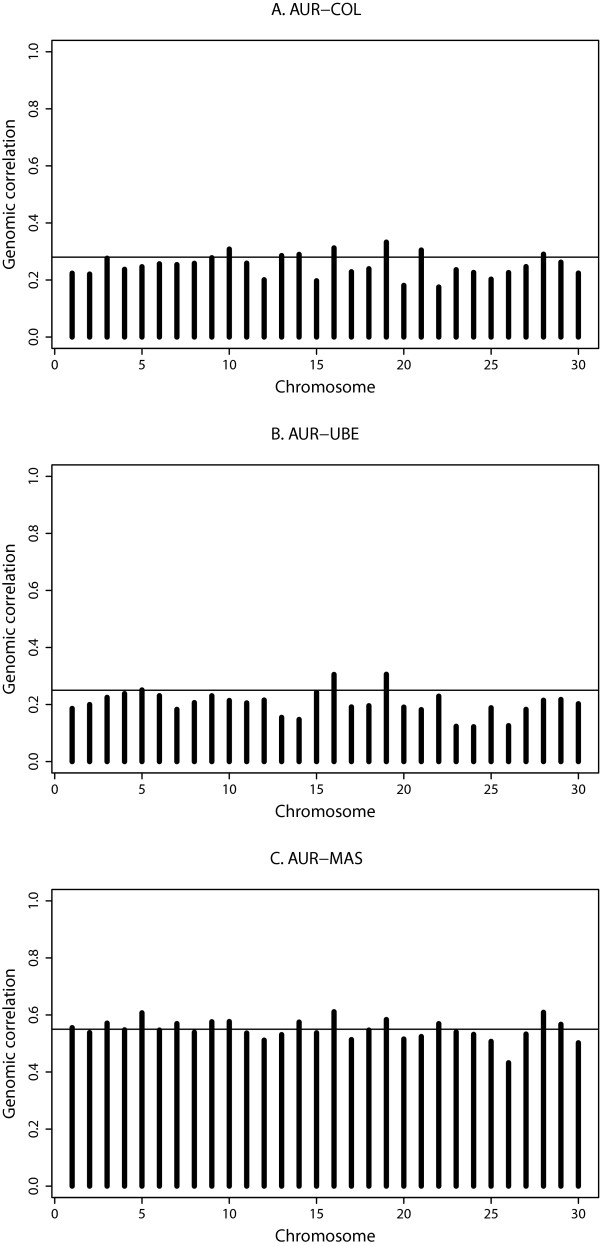
**Examples of genomic correlations per chromosome.** AUR: *Staph. aureus*; COL: *E. coli*; UBE: *Strep. uberis*; MAS: general mastitis; horizontal lines indicate genome-wide genomic correlations.

In Figure 5, three examples are shown to illustrate the differences in chromosome-wise genomic correlations between pair-wise trait combinations with *Staph. aureus*. Differences between chromosomes were still noticeable but were far less pronounced compared with the chromosome-wise genomic covariances. Similar to the chromosome-wise GPV, some chromosomes had larger genomic correlations than expected based on the genome-wide genomic correlation. This could indicate the presence of pleiotropic QTL, e.g. BTA16 and 19 for the combination of *Staph. aureus* and *Strep. uberis*. In contrast to the genomic covariances, the genomic correlations did not depend on chromosome size but were similar across chromosomes.

### Region-wise genomic variance and correlation

BTA19 was further investigated as this chromosome showed a clear effect on all traits. Profiles of genomic variances across this chromosome were created by computing posterior variances in half-overlapping blocks of 100 SNP for each trait. This means that one computation was done for blocks with SNP 1–100, 101–200 etc., and a second computation was done for blocks with SNP 1–50, 51–150 etc. and then the values of overlapping blocks were averaged to smooth out blocks of 50 SNP.

**Figure 6:**
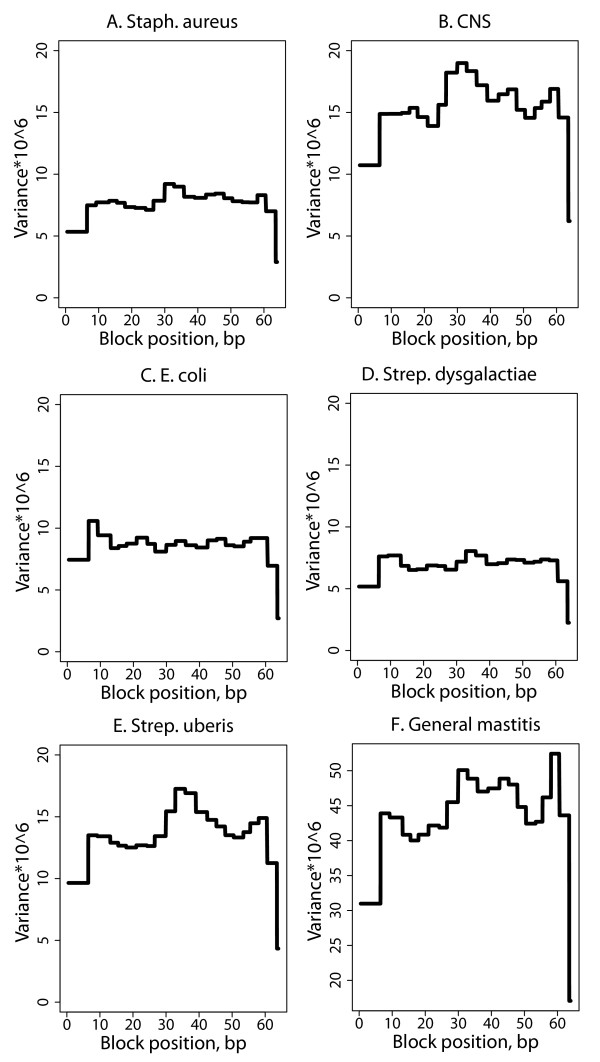
Genomic variance on BTA19 by 50-SNP blocks for the six mastitis traits (A-E).

In general, the genomic variance on BTA19 (Figure 6) was spread across the entire chromosome, except at the chromosome ends, with regions of larger variance than average. The variance patterns differed between traits but a common peak was observed around 35 Mb for the pathogen-specific traits except *E. coli*. This peak was most pronounced for CNS and *Strep. uberis*. Also, a peak was observed around 10 Mb for *E. coli* and *Strep. dysgalactiae*. No clear peaks were observed for general mastitis, possibly because of the composition of this trait.

**Figure 7:**
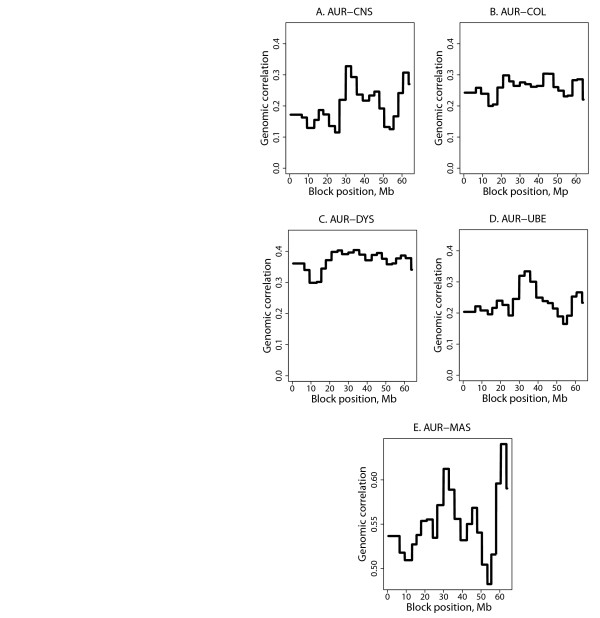
**Genomic correlations on BTA19 by 50-SNP blocks between *Staph. aureus* mastitis and the remaining mastitis traits (A-D).** AUR: *Staph. aureus*; COL: *E. coli*; DYS: *Strep dysgalactiae*, UBE: *Strep. uberis*; MAS: general mastitis.

Establishing the profiles of genomic correlations for BTA19 as described above, revealed the pleiotropic regions along the chromosome. Figure 7 shows such profiles for the genomic correlations between *Staph. aureus* and the other traits. The magnitude of the genomic correlations differed between trait combinations, with the largest genomic correlations obtained between *Staph. aureus* and general mastitis because of their part-whole relationship. The genomic correlation between pathogen-specific traits was highest for *Staph. aureus* and CNS, likely because both are staphylococci. The genomic correlation between *Staph. aureus* and *Strep. dysgalactiae* was also high, while that between *Staph. aureus* and *Strep. uberis* was the lowest, followed by the correlation between *Staph. aureus* and *E. coli*. The positions and widths of the pleiotropic regions differed between trait combinations. For example, a very narrow region around 35 Mb was observed for the genomic correlation between *Staph. aureus* and *Strep. uberis,* while it was much wider around 30 Mb for the genomic correlation between *Staph. aureus* and *E. coli*.

## Discussion

### Whole-genome GPV

The estimated values for GPV express the proportion of the total variance (additive genetic variance + residual variance) of the PM that is explained by the markers and can therefore be related to the heritabilities of the PM. However, because the PM are mean phenotypes, the explained variance in PM is increased relative to heritabilities of individual phenotypes. The GPV estimates were 80% to 87% of the pedigree based estimates of heritability but almost perfectly lined up with each other. Variances explained by markers can be expected to be lower than pedigree-based variances because the markers used may not be in complete LD with the causal polymorphisms [23].

The ranking of the GPV across traits was slightly different from the ranking of heritabilities reported in [1], in which the heritability was lowest for mastitis caused by *Staph. aureus,* followed by *Strep. dysgalactiaeE. coli*, CNS, *Strep. uberis* and general mastitis. In our study, the rankings of *Staph. aureus* and *Strep. dysgalactiae* and CNS and *Strep. uberis* were shifted around. Heritability of general mastitis was up to three times higher than that of pathogen-specific mastitis in [1] while in our case when considering the mean GPV, the highest average GPV was obtained for mastitis caused by CNS. Posterior means of GPV for a trait differed little between the pair-wise analyses of the traits. This indicates that the model is robust and performs well for this parameter. However, it is not clear why we see the difference in ranking between the GPV and traditionally estimated heritabilities. This could be related to data issues such as disease incidences or reliabilities of the PM.

### Whole-genome correlation

The posterior means of the genome-wise genomic correlations were all lower than the traditionally estimated genetic correlations based on pedigree [1,11,24]. Also, the ranking of the genomic correlations among the pathogen-specific mastitis traits was different compared with the pedigree-based results reported in [1]. The genomic correlations between general mastitis and the pathogen-specific mastitis traits were expected to be higher than among the pathogen-specific mastitis traits because of part-whole relationships. We did find higher values for correlations involving general mastitis but compared to [24], who reported values close to unity (0.87 to 0.94) based on a linear pedigree-based sire model, the values found in the present study were much lower. We have no clear explanation for why the genomic correlations are different from the pedigree-based estimates. If all the genomic variance is captured but not all the covariance, this will lead to a lower genomic correlation and vice versa. The overall covariance could be affected by the priors used in the model, which assumed a common covariance across the genome.

### Chromosome-wise GPV

Genomic proportions of the total variance were also estimated per chromosome for all traits. Generally, the magnitude of the GPV was increased with size of the chromosomes. However, some chromosomes had slightly higher GPV than average compared to their size and for other chromosomes, the GPV was a little lower than expected based on size. The dependency on chromosome size can partly be explained by the number of markers on each chromosome. The model assumes a priori the same variances for all SNP, which is in line with the standard assumption in gBLUP [9]. Thus, larger chromosomes, which harbor more SNP, will a priori explain more variance than smaller chromosomes. In contrast to using a mixture prior for the SNP effects, a common prior will equalize the SNP effects across the genome and is less suitable to detect small differences in SNP effects.

Based on the results presented here, higher than expected (based on size) chromosome-wise GPV may indicate the presence of QTL on the chromosome. Chromosomes that explained most variance may also harbor the most important QTL (major genes) affecting the trait. Clear differences in the GPV profiles were observed between the six traits. However, working at the chromosome level is likely not detailed enough to detect clear pathogen-specific regions since no clear distinctions between traits were detected regarding chromosomes which explain more variance than expected, except for BTA19, which had a relatively high GPV for all traits, including general mastitis. This may indicate that this chromosome harbors QTL with a general effect on mastitis resistance.

Information about QTL in the present study could be better compared to traditional QTL methods. In contrast to multi-trait implementations of traditional QTL mapping based on linkage e.g. [25-28], we used a 50 K SNP panel and a simpler association model to perform mapping, which was possible because the marker map is dense. We also modeled all markers simultaneously to circumvent problems with false positive results and double counting of effects from correlated SNP, which was necessary to accumulate whole-genome effects. And finally, we had more animals with genotypes. Thus, our genomic model collected the effects of smaller QTL that may not be detectable by traditional QTL methods. Also, the results shown here were GPV for whole chromosomes, and a single QTL with a large effect in a chromosome may not show clearly in the total chromosome GPV. Possibly, several large QTL may have to be present in a chromosome to show a marked effect on the total chromosome GPV.

In general, the genomic model used here was rather basic, as both a common variance and covariance for SNP effects were applied. It is possible to extend the model to take in account different (co)variances per chromosome or of other defined genomic regions. This would be necessary to more accurately predict effects of QTL affecting one or more traits. Also, there are different ways of defining genomic regions, which is an area that needs further investigation. Below, we discuss how for example this could be done in a rather simple way.

### Chromosome-wise genomic covariances and correlations

The most interesting feature of our multi-trait genomic model is the ability to estimate covariances between defined genomic regions for each trait, which would be more difficult with other approaches. For example, with traditional BLUP estimation, it would be necessary to build 30 genomic relationship matrices and to simultaneously estimate 90 covariance components. Dividing each chromosome into several segments would further increase these numbers and the memory requirements for computation. For the present SNP-based model, the model is run once and relevant parameters are inferred from the MCMC samples. As with the genomic variances, the traditional gBLUP model [9] was extended to a REML version to estimate the covariances from data. One can criticize the prior assumption that SNP contribute equal covariance as being somewhat simplistic but it is a common model used in genomic selection. Our main reason to implement this REML approach in a Bayesian context is that it makes it possible to partition covariances into genome segments. In the posterior distributions of the Bayesian model, deviations appear from the prior expectation of common covariance. We can show this by computing covariances by groups of SNP. Here, the effect of the common prior distribution is that chromosome and genome-segment covariances will be regressed towards the estimated common overall covariance, while deviations in the posterior estimates will be informative to show where the genome contributes more or less covariance.

Interesting chromosomes are indicated by covariances above the average covariance of the genome or chromosome. Chromosomes showing large effects were in some cases much clearer based on covariances than based on GPV. Similar to the chromosome GPV, a clear relationship with chromosome size was also observed for the chromosome covariances.

All chromosome covariances among the pathogen-specific mastitis traits were positive. This indicates that genes that control mammary response towards one pathogen (e.g. release of immune factors a.o.) to a certain degree also control response towards other pathogens. However, it is difficult to interpret the absolute differences between the chromosome covariances because our model pulls these estimates towards a common average. In reality, the chromosome covariances will be more different than shown here, but it would be difficult to estimate variances and covariances for 30 chromosomes in a fully unconstrained way.

Information about chromosome-wise covariances can be useful when a QTL has been found and knowledge about potential effects on other traits is required. One could argue that chromosome covariances between traits vary more when the pathogens are more distantly related or show different infection patterns. For example, the chromosome covariances between *Staph. aureus* and *E. coli* differed much more (higher covariance than expected on more chromosomes) from their expected values (based on chromosome size) than the covariances between *Strep. uberis* and *Strep. dysgalactiae* or covariances between general mastitis and the pathogen-specific mastitis traits. Also, the use of a common prior resulted in lower genome-wide genomic correlations, which quantify the relatedness between two traits.

It is not clear whether our method can be used to identify chromosomes that harbor pleiotropic QTL using the current settings. According to Figure 5, the chromosome-wise genomic correlations plots may provide better information than genomic covariances about chromosomes that harbor pleiotropic QTL. Results for the *Staph. aureus*/*E. coli* combination suggest that BTA16 and 19 are the only chromosomes that harbor QTL affecting mastitis caused by each of these pathogens. However, BTA16 and 19 also seem to be clear candidates for harboring pleiotropic QTL affecting both *Staph*. *aureus* and *Strep. uberis*. Finally, at least five chromosomes (BTA5, 14, 16, 19, and 28) are likely to harbor QTL that affect resistance towards *Staph. aureus* and general mastitis.

A QTL that affects specific mastitis must also affect general mastitis, but it may not be detected, e.g. the specific mastitis may only contribute little to general mastitis. More likely, the QTL detected for general mastitis are QTL affecting the more prevalent and multiple mastitis cases.

### Region-wise genomic variances and correlations

One way to overcome the problem of averaging out QTL for the purpose of QTL mapping may be by splitting the chromosomes up into smaller regions, for example based on neighboring SNP that are in LD with each other. Then, the defined region consists of SNP that are more likely to cluster around potential QTL and the effect is not distorted by many SNP with very small or zero effects. In the present study, this was done in a simple way by computing genomic (co)variances in half-overlapping intervals of 100 SNP on BTA19 because this chromosome was the only chromosome with a clear effect on all traits. This method revealed different variance profiles between the traits. No clear peaks were detected because of the use of a common prior which, as explained above, equals out the variance across the defined regions. The peaks were more pronounced. The use of a mixed prior distribution for the SNP effect may be more appropriate to detect QTL regions. However, to date this method only works well for single-trait analyses and must be further investigated.

The LD between SNP is accounted for when genomic (co)variances are calculated. This means that the total genomic variance may differ from the sum of region-wise or chromosome-wise variance, because the latter would ignore covariance between the parts. In our analysis, these sums of chromosome and region variances were smaller than the total genome-wide variance, indicating presence of negative covariances between the parts.

## Conclusions

The results from the present study show that it is possible to study, genome- and region-wise genomic (co)variances of mastitis resistance traits in dairy cattle using a multivariate genomic model. It was found that larger chromosomes explained more genomic variance than smaller chromosomes due the larger chromosomes having more SNP. Some chromosomes explained more variance than expected according to chromosome size. This could indicate that these chromosomes harbor QTL affecting the traits. Clear differences in variance profiles among the investigated traits were observed, indicating that the mammary response to infections differs between pathogens. All chromosomes explained positive covariances between the traits as a result of the model assumptions. As with the genomic variance, some chromosomes explained more covariance than expected according to their size. This could indicate the presence of pleiotropic QTL. With this methodology and PM as phenotypes, the estimated genomic correlations between the traits were found to be lower than genetic correlations estimated by traditional methods based on pedigree, which indicates that these values are not necessarily comparable. In our model, a rather simple approach was applied to model SNP effects, i.e. a common variance and covariance for the SNP effects. However, the results provide an opportunity to develop this model with different model assumptions, e.g. mixture priors for the SNP effect, which could allow the model to more accurately accommodate differences in (co)variances across the genome.

## Competing interests

The authors declare that they have no competing interests.

## Authors’ contributions

LPS performed the statistical analyses and drafted the manuscript. LJ developed the software for the analyses and gave valuable inputs to the description of methods. LJ, PM, TM and MSL provided valuable suggestions for analyses, interpretation of results and the discussion. All authors were involved in proof reading the manuscript and have approved the final manuscript.
